# Pro-Inflammatory Markers in Persons Living with HIV and HCV: The Role of Opioid Use

**DOI:** 10.3390/pathogens15101021

**Published:** 2026-09-29

**Authors:** Wei-Wen Hsu, Heidi L. Meeds, Janani Madhuravasal Krishnan, Solomon K. Amuzu, Kevin Fedders, Krishna M. Roskin, Matthew Juhascik, John M. Cafardi, Jennifer L. Brown, Caroline Freiermuth, Michael S. Lyons, Kenneth E. Sherman, Jason T. Blackard

**Affiliations:** 1 Division of Biostatistics and Bioinformatics, Department of Biostatistics, Health Informatics and Data Sciences, University of Cincinnati College of Medicine, Cincinnati, OH 45267, USA; hsuwe@ucmail.uc.edu; 2 Department of Internal Medicine, University of Cincinnati College of Medicine, Cincinnati, OH 45267, USA; meedshi@ucmail.uc.edu (H.L.M.); jananimk@ucmail.uc.edu (J.M.K.); amuzusk@mail.uc.edu (S.K.A.); fedderkt@mail.uc.edu (K.F.); shermake@ucmail.uc.edu (K.E.S.); 3 Divisions of Biomedical Informatics and Immunobiology, Cincinnati Children’s Hospital Medical Center, Cincinnati, OH 45229, USA; krishna.roskin@cchmc.org; 4 Department of Pediatrics, University of Cincinnati College of Medicine, Cincinnati, OH 45229, USA; 5 Miami Valley Regional Crime Laboratory/Montgomery County Coroner’s Office, Dayton, OH 45402, USA; juhascikm@mcohio.org; 6 Division of Infectious Diseases, Department of Internal Medicine, The Christ Hospital and the Lindner Research Institute, Cincinnati, OH 45219, USA; john.cafardi@thechristhospital.com; 7 Department of Psychological Sciences, Purdue University, West Lafayette, IN 47907, USA; brow2319@purdue.edu; 8 Center for Addiction Research, University of Cincinnati College of Medicine, Cincinnati, OH 45267, USA; eadyce@ucmail.uc.edu; 9 Department of Emergency Medicine, University of Cincinnati College of Medicine, Cincinnati, OH 45219, USA; 10 Department of Emergency Medicine, Yale School of Medicine, New Haven, CT 06519, USA; michael.lyons@osumc.edu; 11 Massachusetts General Hospital, Harvard Medical School, Wang Ambulatory Care Center-5, Boston, MA 02114, USA

**Keywords:** HIV, HCV, fentanyl, opioids, cytokines, inflammation, apoptosis, immune activation

## Abstract

Background: The United States continues to experience a major drug epidemic driven by synthetic opioids, particularly fentanyl. Emerging evidence suggests that opioids alter immune responses and viral pathogenesis. This study evaluated inflammatory and apoptotic markers among individuals living with HIV and/or hepatitis C virus (HCV) infection in the context of opioid/fentanyl exposure. Methods: Whole blood was collected from adults with HIV, HCV, and/or opioid use disorder (OUD), along with healthy controls. Plasma toxicology screening for illicit substances was performed using LC-MS/MS. Inflammatory cytokines and chemokines (IL-6, IL-8, SDF-1α, MIP-1α, MIP-1β, RANTES, and TNF-α) were measured by Luminex, and M30 was measured by ELISA. Biomarkers were compared using the Kruskal–Wallis test, with multivariable regression used to identify independent associations. Results: Among 98 participants, 37.8% had OUD, 53.9% had a history of injection drug use, and 95.9% of toxicology-confirmed opioid users tested positive for fentanyl. Participants with HIV had higher RANTES and lower M30 levels, whereas participants with HCV had higher IL-8, MIP-1β, TNF-α, and M30 levels and lower RANTES. Multivariable analysis identified independent associations of IL-8 with HIV and HCV status, SDF-1α with injection drug use and recent fentanyl exposure, and MIP-1β with HCV status. The number of substances detected was not independently associated with inflammatory marker levels. Conclusions: HIV and HCV were associated with distinct inflammatory and apoptotic profiles in the setting of widespread fentanyl exposure, suggesting that chronic viral infection and opioid exposure contribute to immune dysregulation and may influence disease progression.

## 1. Introduction

The United States opioid epidemic continues to evolve rapidly, with synthetic opioids such as fentanyl dominating the illicit drug supply [1,2]. Fentanyl-related overdose deaths have risen dramatically over the last decade and disproportionately impact vulnerable populations, including persons living with HIV (PLWH) and/or hepatitis C virus (HCV) infection [3]. Injection drug use remains a major risk factor for HIV and HCV transmission, while polysubstance use further complicates disease management and clinical outcomes.

Beyond transmission risk, opioids are increasingly recognized as important modulators of immune function and viral pathogenesis. Experimental studies have demonstrated that opioids alter innate and adaptive immune responses, impair interferon signaling, modulate cytokine production, and influence viral replication [4,5,6]. We previously demonstrated that fentanyl enhances HIV replication in lymphocyte cell lines, macrophages, and peripheral blood mononuclear cells [7,8,9,10]. Additional evidence suggests that opioids may enhance hepatitis virus replication and suppress antiviral immune pathways, including altered interferon signaling and innate immune responses [4]. Chronic inflammation and immune activation contribute substantially to morbidity and mortality in both HIV and HCV infection. Persistent inflammatory signaling is associated with cardiovascular disease, liver fibrosis, neurocognitive dysfunction, metabolic complications, and accelerated aging in PLWH [11,12]. Similarly, chronic HCV infection induces sustained hepatic inflammation, cytokine dysregulation, and hepatocyte apoptosis that contribute to fibrosis progression and liver injury [13].

Chemokines such as RANTES (CCL5), MIP-1β, and SDF-1α (CXCL12) are particularly relevant in viral infection because they regulate leukocyte trafficking and HIV co-receptor interactions [14,15]. Pro-inflammatory cytokines such as IL-8 and TNF-α also contribute to hepatic inflammation and immune activation in chronic viral infection [16]. In HIV infection, persistent elevation of pro-inflammatory cytokines, including TNF-α, IL-6, and IL-8, contributes to chronic immune activation, which is a major driver of disease progression and non-AIDS comorbidities despite effective antiretroviral therapy. Furthermore, increased apoptosis of immune and non-immune cells has been implicated in HIV-associated immune dysfunction and tissue injury [17,18]. Cytokeratin-18 fragments—measured as M30—are established biomarkers of caspase-mediated apoptosis and have been associated with hepatocyte injuryand liver disease [19,20,21]. Understanding how opioid use influences these pathways is critical for defining mechanisms of disease progression.

Given the increasing prevalence of fentanyl exposure among people living with HIV and HCV, the present study characterizes systemic inflammatory and apoptotic marker profiles in individuals with HIV and/or HCV infection and evaluated associations with opioid use.

## 2. Methods

### 2.1. Study Population

Adults presenting to the University of Cincinnati Medical Center Emergency Department between August 2019 and February 2024 were prospectively enrolled. Participants included individuals living with HIV and/or HCV infection with or without opioid exposure, as well as healthy HIV- and HCV-negative controls without use of opioid. Participants completed surveys regarding opioid use history, injection drug use, and demographic characteristics. Medical chart review was conducted to determine infectious disease and opioid use disorder diagnostic information. Inclusion criteria included age ≥ 18 years and the ability to provide informed consent.

### 2.2. Toxicology Screening

Opioid exposure was characterized using complementary clinical, self-reported and toxicological measures. Participants completed surveys regarding prior opioid use, fentanyl exposure, injection drug use, and drug use within the previous 24 h. OUD information was obtained through medical chart review. Plasma collected at enrollment was screened using targeted liquid chromatography-tandem mass spectrometry (LC-MS/MS) [22] for opioids, fentanyl analogs, cannabinoids, stimulants, benzodiazepines, and other prescription or illicit compounds. Toxicology results were interpreted as evidence of recent exposure and were not considered measures of the frequency, duration, or chronicity of opioid use.

### 2.3. Cytokine and Apoptosis Marker Analysis

Plasma concentrations of IL-6, IL-8, SDF-1α, MIP-1α, MIP-1β, RANTES, and TNF-α were quantified using multiplex Luminex cytokine assays (Luminex/DiaSorin, Austin, TX, USA). Plasma M30 concentrations were measured using the M30 Apoptosense^®^ ELISA (PEVIVA, BioNordika AB, Stockholm, Sweden), according to the manufacturer’s instructions.

### 2.4. Statistical Analysis

Median biomarker levels were compared by HIV and HCV status using the Kruskal–Wallis test. Because multiple cytokines/chemokines were compared across HIV or HCV status, we controlled the false discovery rate using the Benjamini–Hochberg procedure, which was applied separately within each comparison (HIV+ vs. HIV− and HCV+ vs. HCV−). A two-sided False Discovery Rate (FDR)-adjusted *p*-value was reported. Given the exploratory nature of this pilot study, markers with adjusted *p*-values approaching this threshold were also examined descriptively as candidates for future confirmatory work. Multivariable regression models were used to evaluate associations between log_10_-transformed cytokine/chemokine/M30 levels and demographic variables, HIV status, HCV status, non-fentanyl opioid exposure, fentanyl exposure, and injection drug use. Variance Inflation Factor (VIF) analyses were also performed to examine possible collinearity issues. Statistical significance was defined as *p* < 0.05.

## 3. Results

### 3.1. Participant Characteristics

A total of 98 participants were evaluated for inflammatory marker analyses. Demographic and clinical characteristics are summarized in Table 1. The overall cohort had a mean age of 44.1 years. Approximately 75.5% of participants were male, while 24.5% were female. The majority identified as White (64.3%), with African American participants comprising 35.7% of the cohort. Among the study population, 37.8% had a diagnosis of opioid use disorder (OUD), and 53.9% reported a history of injection drug use. Opioid exposure was highly prevalent, with 55.1% reporting prior opioid use. Among participants with opioid exposure, 95.9% had fentanyl exposure, highlighting the predominance of fentanyl within the contemporary opioid supply. Participants with HCV demonstrated higher frequencies of OUD and injection drug use compared with individuals without HCV. Elevated aspartate aminotransferase (AST) and alanine aminotransferase (ALT) values were also observed among participants who were HCV-positive, consistent with ongoing hepatic inflammation.

### 3.2. Inflammatory and Apoptotic Marker Profiles

Inflammatory marker profiles stratified by HIV and HCV status are summarized in Table 2.

Overall, substantial variability in inflammatory and apoptotic marker levels was observed throughout the cohort, reflecting heterogeneous immune activation states associated with chronic viral infection and substance exposure.

To further characterize the cohort by combined HIV/HCV status, participants with complete HIV and HCV data were stratified into four groups: HIV−/HCV− (n = 10), HIV+/HCV− (n = 39), HIV−/HCV+ (n = 18), and HIV+/HCV+ (n = 22). Demographic, clinical, and substance use characteristics of these groups are presented in Supplementary Table S1. Corresponding inflammatory and apoptotic biomarker profiles across the four groups are presented in Supplementary Table S2. Nine participants with incomplete HIV or HCV status were excluded from these subgroup analyses. Given the small and unequal subgroup size, these analyses were considered exploratory.

### 3.3. HIV-Associated Inflammatory Profiles

PLWH demonstrated significantly elevated RANTES levels compared with HIV-negative participants (363.1 vs. 174.9 pg/mL; *p* = 0.014). In contrast, M30 levels were significantly lower among PLWH (84.4 vs. 114.7 U/L; *p* = 0.009).

Although they were not significant, PLWH also demonstrated trends toward lower IL-6 levels and higher SDF-1α concentrations compared with HIV-negative individuals.

### 3.4. HCV-Associated Inflammatory Profiles

HCV-positive participants demonstrated higher levels of several inflammatory cytokines and chemokines compared with HCV-negative participants. IL-8 levels were significantly higher among HCV-positive participants compared with HCV-negative individuals (9.6 vs. 6.3 pg/mL; *p* = 0.023). Elevated MIP-1β levels were also observed among HCV-positive participants (25.7 vs. 18.4 pg/mL; *p* = 0.029), along with elevated TNF-α levels (17.3 vs. 10.7 pg/mL; *p* = 0.033). M30 levels were significantly elevated among HCV-positive individuals (105.7 vs. 90.0 U/L; *p* = 0.029), consistent with increased hepatocyte apoptosis and hepatic inflammatory injury. Interestingly, RANTES levels were significantly lower among HCV-positive individuals compared with HCV-negative controls (210.5 vs. 363.9 pg/mL; *p* = 0.044), suggesting differential chemokine regulation between HIV and HCV infection. Collectively, these findings suggest that chronic HCV infection is associated with a strongly pro-inflammatory and pro-apoptotic systemic profile.

### 3.5. Multivariable Regression Analyses

Regression analyses evaluating transformed chemokine outcomes are summarized in Table 3. All VIF values ranged from 1.23 to 7.08 (not shown; all < 10, a common liberal cutoff), indicating that collinearity was within acceptable limits. Multivariable regression modeling was performed to better define independent associations between inflammatory marker levels and demographic, clinical, and substance exposure variables while controlling for potential confounding factors. Specifically, the regression models incorporated age, sex, race, HIV status, HCV status, HIV-HCV interaction terms, history of injection drug use, opioid exposure history, and drug exposure within the previous 24 h. Biomarker outcomes were log_10_-transformed prior to analysis to normalize the data distribution and reduce the influence of extreme values.

### 3.6. IL-8 Regression Analysis

Among all evaluated inflammatory markers, IL-8 demonstrated independent associations with chronic viral infection. HIV-positive status (coefficient estimate = 0.363; *p* = 0.038) and HCV-positive status (coefficient estimate = 0.518; *p* = 0.036) were associated with IL-8 concentrations after adjustment for covariates. Importantly, a statistically significant HIV-HCV interaction term was identified for IL-8 (coefficient estimate = −0.651; *p* = 0.008), suggesting that the combined effects of HIV and HCV coinfection may influence inflammatory signaling differently (i.e., lower IL-8) than either infection alone. This interaction may reflect complex immune regulatory mechanisms in coinfected individuals, potentially involving altered cytokine production, immune exhaustion, or chronic inflammatory adaptation.

Neither opioid exposure history nor recent fentanyl exposure demonstrated independent associations with IL-8 levels after adjustment for viral infection status and demographic variables. Similarly, age, sex, race, and recent injection drug use were not significantly associated with IL-8 concentrations in the adjusted model.

### 3.7. SDF-1α Regression Analysis

SDF-1α (CXCL12) demonstrated distinct associations with injection drug use and fentanyl exposure. History of injection drug use remained independently associated with elevated SDF-1α levels after adjustment for covariates (coefficient estimate = 0.142; *p* = 0.027). Fentanyl exposure within the previous 24 h was also marginally associated with elevated SDF-1α concentrations (coefficient estimate = 0.136; *p* = 0.048). These findings suggest that fentanyl exposure and factors associated with injection drug use may influence SDF-1α-mediated inflammatory and endothelial signaling pathways. In addition to repeated vascular injury from injection practices, injection drug use is associated with recurrent bacterial infections, exposure to additional blood-borne pathogens, and chronic immune stimulation, all of which may contribute to altered CXCL12 signaling. Elevated SDF-1α levels may therefore reflect a combination of endothelial injury, systemic inflammation, and compensatory tissue repair responses associated with opioid exposure and injection drug use.

Interestingly, HIV status, HCV status, and the HIV-HCV interaction term were not independently associated with SDF-1α concentrations after adjustment for covariates. Similarly, demographic variables including age, sex, and race did not demonstrate significant independent associations.

### 3.8. MIP-1β Regression Analysis

MIP-1β remained significantly associated with HCV status (coefficient estimate = 0.387; *p* = 0.008) and HIV-HCV interaction term (coefficient estimate = −0.301; *p* = 0.034), after multivariable adjustment, suggesting that chronic HCV infection and HIV-HCV co-infection exerts an important independent effect on chemokine dysregulation. The significant HIV-HCV interaction term was specifically supporting the concept that coinfection may differentially influence immune activation and chemokine signaling pathways. Although HIV status alone was not independently associated with MIP-1β levels, a trend towards elevated concentrations was observed among PLWH. Race also demonstrated a near-significant association with MIP-1β levels (*p* = 0.056), suggesting possible demographic influences on inflammatory responses that may warrant additional investigation in larger cohorts. Opioid exposure history, recent opioid use, and recent fentanyl exposure were not statistically associated with MIP-1β concentrations after adjustment for viral infection status and demographic variables.

### 3.9. Overall Regression Findings

Collectively, the regression analyses demonstrated that HIV and HCV infection status were independently associated with several systemic inflammatory markers, particularly IL-8 and MIP-1β, highlighting the contribution of chronic viral infection to inflammatory signaling pathways. In contrast, apart from the associations observed between SDF-1α and both injection drug use history and recent fentanyl exposure, neither non-fentanyl opioid exposure nor cumulative substance burden was independently associated with most inflammatory or apoptotic markers after adjustment for covariates. The association between SDF-1α and injection drug use suggests that factors associated with injection practices, including vascular injury, recurrent infections, and opioid-related inflammatory pathways, may influence CXCL12-mediated immune signaling independent of viral infection. Although fentanyl and polysubstance exposure were highly prevalent within this cohort, the cross-sectional nature of the study may not fully capture the cumulative effects of chronic opioid exposure on immune dysregulation. Additional longitudinal and mechanistic studies are warranted to further define the complex interactions among viral infection, fentanyl exposure, polysubstance use, and inflammatory signaling pathways.

## 4. Discussion

The present study provides insight into systemic inflammatory and apoptotic profiles among individuals living with HIV and/or HCV in the setting of widespread fentanyl exposure and polysubstance use. One of the most striking findings was the near ubiquity of fentanyl exposure among opioid-positive individuals. Nearly all participants reporting opioid exposure had fentanyl detected, underscoring the dominance of fentanyl within the current illicit opioid supply. These findings align with national epidemiologic data demonstrating that fentanyl now accounts for the majority of opioid-related overdose deaths in the United States [1,2,3].

The cohort also demonstrated extensive polysubstance exposure, reflecting increasingly complex drug use patterns. Prior studies have demonstrated that concurrent exposure to opioids, stimulants, cannabinoids, and sedatives may produce additive effects on immune dysregulation, neuroinflammation, and systemic injury [23]. Polysubstance exposure may therefore contribute substantially to disease progression in persons living with chronic viral infection. Distinct inflammatory signatures were associated with HIV and HCV infection. PLWH demonstrated elevated RANTES levels compared with HIV-negative controls. RANTES (CCL5) is a key chemokine involved in HIV pathogenesis because it competes for CCR5 receptor binding and influences leukocyte trafficking [14]. Elevated RANTES levels may represent persistent immune activation despite viral suppression and antiretroviral therapy. In contrast, participants with HCV demonstrated significantly elevated IL-8, MIP-1β, TNF-α, and M30 levels. Chronic HCV infection is characterized by sustained hepatic inflammation and immune activation, and elevated IL-8 and TNF-α have previously been associated with fibrosis progression and hepatocellular injury [13,16]. Increased M30 levels further support enhanced hepatocyte apoptosis and ongoing hepatic injury among HCV-positive individuals [21].

An intriguing finding was the contrasting regulation of RANTES between HIV and HCV infection. While PLWH demonstrated elevated RANTES levels, HCV-positive participants demonstrated reduced RANTES concentrations relative to HCV-negative individuals. These opposing patterns suggest that HIV and HCV differentially regulate chemokine signaling pathways and immune cell trafficking.

Regression analyses demonstrated significant associations between IL-8 and both HIV and HCV status, suggesting that IL-8 may represent a shared inflammatory mediator linking chronic viral infection with persistent immune activation. SDF-1α was independently associated with both injection drug use history and recent fentanyl exposure, indicating that opioid exposure and vascular injury associated with injection drug use may influence CXCL12 signaling pathways. In contrast, cumulative substance burden was not independently associated with inflammatory marker levels after adjustment for covariates. These findings suggest that chronic viral infections, such as HIV and HCV, may exert a stronger influence on systemic inflammatory signatures than the number of substances detected at a single time point. Alternatively, acute toxicology screening may not adequately capture the duration, frequency, or cumulative effects of substance exposure that contribute to immune dysregulation. Experimental studies have shown that opioids suppress interferon signaling, impair immune cell function, alter cytokine production, and enhance viral replication [4,5,6,7,8,9]. These mechanisms may contribute to chronic immune activation, viral persistence, and tissue injury among persons exposed to opioids and fentanyl. This study has several strengths, including comprehensive toxicology screening, inclusion of clinically relevant inflammatory and apoptotic markers, and evaluation of real-world fentanyl exposure patterns However, several limitations should also be acknowledged. The cross-sectional design limits causal inference, and inflammatory markers were measured at a single time point. In addition, because participants were recruited in an emergency department setting, acute illness, infection, physiological stress, and/or other clinical conditions present at the time of enrollment may have influenced circulating inflammatory biomarker levels. These factors were not systematically incorporated into the present analysis; therefore, some of the observed biomarker differences between participant groups may reflect differences in acute clinical status at the time of sampling in addition to chronic viral infection and substance exposure. Although toxicology provides an objective measure of recent exposure, it cannot determine the frequency, duration, or cumulative intensity of opioid use. Detailed longitudinal measures of opioid/fentanyl use were not systematically available for all participants and therefore could not be incorporated into the current analyses. Future studies incorporating repeated toxicology assessments together with detailed measures of frequency, duration, and cumulative exposure will be important for defining the relationship between chronic fentanyl exposure and immune dysregulation. The sample size was modest, particularly for analyses stratified by combined HIV/HCV status. Stratification into four mutually exclusive groups resulted in small and unequal subgroup sizes limiting statistical power and the precision of subgroup-level inference. Furthermore, the study was not originally designed or powered for four-group comparisons. Therefore, these subgroup analyses should be considered exploratory, and larger studies are needed to more comprehensively evaluate inflammatory and apoptotic profiles across HIV/HCV single-infection and coinfection groups. After the FDR correction for multiple comparisons, no cytokine/chemokine was significant at the level of 0.05, although several fell just slightly above this 0.05 threshold. Given the small sample and the non-normal distribution of the analytes, a possible explanation for those borderline results is due to the limited power rather than a true absence of effect. The present findings should therefore be interpreted as exploratory and warrant confirmation in larger, adequately powered studies. Furthermore, the study population was recruited from a single geographic region and may not fully represent broader populations of people living with HIV and/or HCV or individuals with differing patterns of substance use. Although antiviral treatment status was available for a subset of participant, detailed information regarding specific antiretroviral regimens or direct acting agents for HCV, treatment duration, adherence, and treatment response was not available consistently for incorporation into the multivariable analyses. Because antiviral therapies can influence immune activation and inflammatory biomarker profiles, residual confounding related to treatment and underlying disease status cannot be excluded. Therefore, the observed associations between HIV and HCV status and inflammatory biomarkers should be interpreted with consideration of potential differences in antiviral treatment and disease status among participants. Detailed measures of alcohol and tobacco exposure—including frequency, quantity, and timing relative to blood collection—were not systematically available for the entire cohort. Because both exposures may influence systemic inflammatory responses, residual confounding related to alcohol and tobacco use cannot be excluded. The enrollment period also spanned the COVID-19 pandemic and substantial changes in the illicit opioid supply. Pandemic-associated changes in healthcare utilization, psychosocial stress, pattens of substance use and drug availability may therefore have introduced temporal heterogeneity that was not specifically captured in the present analyses. Consequently, the potential influence of these temporal factors on inflammatory biomarker profiles cannot be completely excluded. An additional limitation is that the present study evaluated circulating inflammatory and apoptotic biomarkers and primarily reflects systemic immune activation rather than tissue-specific inflammatory responses. Tissue samples were not collected, precluding direct assessment of compartmentalized immune responses. This distinction may be particularly relevant in HIV infection, where disruption of the gastrointestinal mucosal barrier, local immune activation, and microbial translocation can contribute to persistent systemic inflammation even during antiretroviral therapy. Substance use may further influence mucosal integrity and tissue-specific immune responses. Similarly, in HCV infection, inflammatory and apoptotic processes occurring within the liver may not be fully represented by circulating cytokine, chemokine, or M30 concentrations. Therefore, the peripheral blood biomarker profiles observed in this study should not be interpreted as direct measures of inflammation within the gastrointestinal tract, liver, or other tissue compartments. Future studies incorporating paired peripheral blood- and tissue-based measurements would help determine how systemic inflammatory profiles relate to compartment-specific immune dysregulation in the context of HIV, HCV, and opioid exposure. Nevertheless, these findings provide important translational insights into immune dysregulation among persons living with HIV and/or HCV in the era of fentanyl predominance. Future mechanistic and longitudinal studies are warranted to better define how fentanyl and polysubstance exposure influence antiviral immunity, inflammatory signaling, and disease progression.

## 5. Conclusions

Distinct inflammatory and apoptotic profiles were observed among individuals living with HIV and/or HCV within an emergency care setting. HIV was characterized by higher RANTES and lower M30 levels, whereas HCV was associated with higher IL-8, MIP-1β, TNF-α, and M30 levels and lower RANTES. Multivariable analyses identified associations of IL-8 with HIV and HCV status and of SDF-1α with injection drug use and recent fentanyl exposure. These findings highlight distinct patterns of systemic inflammatory and apoptotic activity associated with chronic viral infection and substance exposure in this cohort. However, because participants were recruited in an emergency department setting, acute illness, infection, physiological stress, and other clinical conditions present at the time of sampling may have influenced circulating biomarker levels. Larger longitudinal studies, including cohorts evaluated outside acute emergency care settings, are needed to determine whether these observed biomarker profiles persist over time and are associated with subsequent disease progression.

## Figures and Tables

**Table 1:** Characteristics of study participants.

	HIV Status		HCV Status		
Variable	Positive (n = 69)	Negative (n = 28)	Positive (n = 40)	Negative (n = 50)	Overall Cohort (n = 98)
Age, mean (SD)	43 (12.7)	43.4 (12.1)	41.5 (9.9)	46.9 (14.2)	44.1 (12.4)
Sex (n, %)					
Male	57 (82.6%)	17 (60.7%)	28 (70.0%)	39 (78.0%)	74 (75.5%)
Female	12 (17.4%)	11 (39.3%)	12 (30.0%)	11 (22.0%)	24 (24.5%)
Race (n, %)					
White	43 (62.3%)	20 (71.4%)	33 (82.5%)	24 (48.0%)	63 (64.3%)
African American	26 (37.7%)	8 (28.6%)	7 (17.5%)	26 (52.0%)	35 (35.7%)
Currently receiving HIV treatment (n, %)					
Yes	49 (73.1%)	-	16 (72.7%)	27 (61.4%)	49 (66.2%)
No	18 (26.9%)	-	6 (27.3%)	17 (38.6%)	25 (33.8%)
Currently receiving hepatitis C treatment (n, %)					
Yes	2 (3.3%)	0 (0%)	2 (5.1%)	-	2 (2.4%)
No	58 (96.7%)	23 (100%)	37 (94.9%)	-	82 (97.6%)
Hepatitis C genotype (n, %)					
Genotype 1 (1a/1b)	6 (28.6%)	5 (29.4%)	9 (25.7%)	-	11 (29.0%)
Genotype 2	2 (9.5%)	1 (5.9%)	3 (8.6%)	-	3 (7.9%)
Genotype 3	4 (19.1%)	2 (11.8%)	6 (17.1%)	-	6 (15.8%)
Genotype 4	1 (4.8%)	1 (5.9%)	2 (5.7%)	-	2 (5.3%)
unknown	8 (38.1%)	8 (47.1%)	15 (42.9%)	-	16 (42.1%)
OUD diagnosis (n, %)					
Yes	23 (33.3%)	14 (50.0%)	29 (72.5%)	4 (8.0%)	37 (37.8%)
No	46 (66.7%)	14 (50.0%)	11 (27.5%)	46 (92.0%)	61 (62.2%)
Drug use ever (n, %)					
Injection drugs	29 (47.5%)	19 (70.4%)	36 (94.7%)	9 (20.5%)	48 (53.9%)
Opioids	30 (49.2%)	19 (70.4%)	36 (97.3%)	10 (22.2%)	49 (55.1%)
Fentanyl	30 (48.4%)	18 (66.7%)	36 (94.7%)	9 (20.0%)	48 (53.3%)
Drug use in last 24 h (n, %)					
Injection drugs	14 (21.9%)	13 (48.2%)	23 (60.5%)	2 (4.3%)	27 (29.4%)
Opioids	16 (25.4%)	13 (48.2%)	24 (64.9%)	3 (6.4%)	29 (31.9%)
Fentanyl	15 (23.4%)	12 (44.4%)	24 (63.2%)	2 (2.13%)	27 (29.4%)
The most recent value (median, Q1–Q3)					
CD4 cell count	368 (160.4–643)	-	379.5 (247.7–652.0)	307 (126.0–466.8)	368 (160.4–643.0)
ALT value	21.5 (13–40)	30 (18–43)	34.5 (19–75)	18 (13–38)	22 (15.0–41.5)
AST value	25 (19–41)	32 (24–46)	36 (19–86)	25 (19–32)	26.5 (19.0–42.5)
Albumin value	4.1 (3.6–4.5)	3.9 (3.4–4.0)	3.9 (3.4–4.2)	4 (3.5–4.5)	3.9 (3.5–4.4)
Platelet count	209 (161–251)	260 (228–293)	239 (164–286)	220 (161–274)	232 (162–274)

**Table 2:** Cytokine/Chemokines/M30 values by HIV and HCV status.

Cytokine/Chemokine	HIV Status		Kruskal–Wallis Test	HCV Status		Kruskal–Wallis Test	Overall Cohort (n = 98)
	Positive (n = 69)	Negative (n = 28)		Positive (n = 40)	Negative (n = 50)		
	Median (Q1–Q3)	Median (Q1–Q3)	*p*-Value (FDR adj *p*)	Median (Q1–Q3)	Median (Q1–Q3)	*p*-Value (FDR adj *p*)	Median (Q1–Q3)
IL-6 pg/mL	3.895 (1.460–8.840)	5.550 (2.285–18.140)	0.079 (0.211)	5.055 (1.485–12.408)	3.140 (1.368–7.688)	0.160 (0.213)	4.255 (1.505–9.305)
IL-8 pg/mL	7.195 (4.255–15.855)	8.440 (4.675–12.775)	0.595 (0.793)	9.608 (5.485–20.903)	6.343 (3.758–13.693)	0.023 * (0.066)	7.695 (4.280–14.660)
SDF1alpha	790.5 (684.500–954.195)	701.730 (630.535–868.775)	0.160 (0.320)	799.345 (634.348–970.875)	750.943 (650.578–847.428)	0.323 (0.369)	773.805 (644.065–921.285)
Mip-1alpha	7.610 (4.002–12.630)	7.250 (3.653–20.000)	0.890 (0.890)	8.788 (3.827–17.153)	6.680 (3.937–13.488)	0.458 (0.458)	7.425 (3.937–14.570)
Mip-1beta	20.730 (15.200–28.565)	25.895 (15.760–32.365)	0.419 (0.670)	25.708 (18.123–32.495)	18.383 (14.915–27.183)	0.029 * (0.066)	20.840 (15.525–29.655)
RANTES	363.050 (171.220–584.160)	174.890 (76.375–361.895)	0.014 * (0.056)	210.540 (80.868–499.935)	363.940 (173.055–554.855)	0.044 * (0.070)	299.740 (150.600–559.470)
TNFalpha	15.185 (9.505–24.450)	12.900 (10.185–30.915)	0.724 (0.827)	17.290 (10.688–31.660)	10.718 (8.505–20.673)	0.033 * (0.066)	13.325 (9.505–25.435)
M30 U/L	84.427 (72.618–108.422)	114.746 (93.734–136.012)	0.009 * (0.056)	105.668 (79.654–209.438)	90.049 (70.775–114.817)	0.029 * (0.066)	96.105 (74.932–118.935)

*: the raw *p*-value < 0.05 and 0.05 < FDR adj. *p*-value < 0.10.

**Table 3:** Regression models for transformed chemokine outcomes.

	IL-8 pg/mL			SDF1alpha			Mip-1beta		
Covariates	Coefficient Estimate	*t*-Test *p*-Value	95% Confidence Interval	Coefficient Estimate	*t*-Test *p*-Value	95% Confidence Interval	Coefficient Estimate	*t*-Test *p*-Value	95% Confidence Interval
Age	0.000	0.994	(−0.010, 0.010)	0.001	0.639	(−0.002, 0.004)	−0.001	0.815	(−0.006, 0.005)
Sex	0.029	0.807	(−0.206, 0.264)	0.014	0.710	(−0.062, 0.091)	0.082	0.239	(−0.056, 0.220)
Race	−0.139	0.234	(−0.371, 0.092)	−0.016	0.672	(−0.091, 0.059)	−0.133	0.056	(−0.268, 0.003)
HIV status	0.363	0.038 *	(0.021, 0.706)	0.055	0.330	(−0.057, 0.166)	0.161	0.113	(−0.039, 0.362)
HCV status	0.518	0.036 *	(0.035, 1.002)	−0.060	0.452	(−0.217, 0.098)	0.387	0.008 *	(0.103, 0.671)
HIV and HCV interaction	−0.651	0.008 *	(−1.124, −0.178)	−0.036	0.644	(−0.190, 0.118)	−0.301	0.034 *	(−0.579, −0.023)
Drug use ever—injection drugs	0.123	0.526	(−0.262, 0.508)	0.142	0.027 *	(0.017, 0.267)	−0.115	0.312	(−0.341, 0.111)
Drug use ever—opioids + fentanyl	0.003	0.988	(−0.389, 0.395)	0.018	0.783	(−0.110, 0.145)	0.011	0.927	(−0.219, 0.240)
Drug use in last 24 h—injection drugs	−0.058	0.737	(−0.403, 0.287)	−0.051	0.370	(−0.163, 0.061)	−0.007	0.943	(−0.209, 0.195)
Drug use in last 24 h—opioids	0.057	0.749	(−0.300, 0.415)	−0.097	0.101	(−0.213, 0.019)	0.091	0.390	(−0.119, 0.301)
Drug use in last 24 h—fentanyl	−0.005	0.981	(−0.419, 0.409)	0.136	0.048	(0.001, 0.271)	−0.139	0.256	(−0.382, 0.104)

*: *p*-value < 0.05.

## Data Availability

The original contributions presented in this study are included in the article. Further inquiries can be directed to the corresponding author.

## Supplementary Materials

The following supporting information can be downloaded at: https://www.mdpi.com/article/10.3390/pathogens15101021/s1, Table S1: Characteristics of study participants by combined HIV/HCV status; Table S2: Inflammatory and apoptotic biomarker profiles by combined HIV/HCV status.
