# Supplementary material for: Pro-Inflammatory Markers in Persons Living with HIV and HCV: The Role of Opioid Use
Source: Pathogens. 2026 Sep 29;15(10):1021. doi: 10.3390/pathogens15101021 (PMC13708061; doi:10.3390/pathogens15101021)
Supplement: Supplementary file 1 [file pathogens-15-01021-s001.zip › Supplementary Table S1.pdf]

Supplementary Table S1. Characteristics of study participants by combined HIV/HCV status.

Variable	HIV- & HCV- (n = 10)	HIV+ & HCV- (n = 39)	HIV- & HCV+ (n = 18)	HIV+ & HCV+ (n = 22)
Age, mean (SD)	40.5 (16.4)	48.9 (13.3)	45.0 (9.2)	38.5 (9.8)
Sex (n, %)				
Male	6 (60.0%)	33 (84.6%)	11 (61.1%)	17 (77.3%)
Female	4 (40.0%)	6 (15.4%)	7 (38.9%)	5 (22.7%)
Race (n, %)				
White	5 (50.0%)	19 (48.7%)	15 (83.3%)	18 (81.8%)
African American	5 (50.0%)	20 (51.3%)	3 (16.7%)	4 (18.2%)
Currently receiving HIV treatment (n, %)				
Yes	-	27 (73.0%)	-	16 (72.7%)
No	-	10 (27.0%)	-	6 (27.3%)
Currently receiving hepatitis C treatment (n, %)				
Yes	-	-	0 (0.0%)	2 (9.1%)
No	-	-	17 (100.0%)	20 (90.9%)
Hepatitis C genotype (n, %)				
Genotype 1 (1a/1b)	-	-	5 (29.4%)	4 (22.2%)
Genotype 2	-	-	1 (5.9%)	2 (11.1%)
Genotype 3	-	-	2 (11.8%)	4 (22.2%)
Genotype 4	-	-	1 (5.9%)	1 (5.6%)
unknown	-	-	8 (47.1%)	7 (38.9%)
OUD diagnosis (n, %)				
Yes	0 (0.0%)	4 (10.3%)	14 (77.8%)	15 (68.2%)
No	10 (100.0%)	35 (89.7%)	4 (22.2%)	7 (31.8%)
Drug use ever (n, %)				
Injection drugs	1 (11.1%)	8 (23.5%)	18 (100.0%)	18 (90.0%)
Opioids	1 (11.1%)	9 (25.7%)	18 (100.0%)	18 (94.7%)
Fentanyl	1 (11.1%)	8 (22.9%)	17 (94.4%)	19 (95.0%)
Drug use in last 24 hours (n, %)				
Injection drugs	0 (0.0%)	2 (5.4%)	13 (72.2%)	10 (50.0%)
Opioids	1 (11.1%)	2 (5.4%)	12 (66.7%)	12 (63.2%)
Fentanyl	0 (0.0%)	1 (2.7%)	12 (66.7%)	12 (60.0%)
The most recent value (median, Q1-Q3)				
CD4 cell count	-	307.0 (126.0-466.8)	-	379.5 (247.7-652.0)

ALT value	22.5 (16.5-35.0)	18.5 (12.0-38.0)	35.0 (19.0-75.0)	34.0 (19.0-72.0)
AST value	30.0 (22.5-34.0)	25.0 (19.0-32.0)	41.0 (24.0-69.0)	31.0 (19.0-86.0)
Albumin value	3.9 (3.5-4.4)	4.1 (3.6-4.5)	3.8 (3.3-4.0)	4.0 (3.6-4.5)
Platelet count	274.0 (241.0-314.0)	212.0 (155.0-243.0)	258.0 (228.0-288.0)	190.0 (161.0-249.0)

OUD, opioid use disorder; ALT, alanine aminotransferase; AST, aspartate aminotransferase; CD4, cluster of differentiation 4; 9 participants with unknown HIV or HCV status were excluded from the subgroup tables. Cells shown as "-" indicate the characteristic is not applicable to that subgroup (e.g., HIV treatment among HIV-negative participants).
