# Supplementary material for: Pro-Inflammatory Markers in Persons Living with HIV and HCV: The Role of Opioid Use
Source: Pathogens. 2026 Sep 29;15(10):1021. doi: 10.3390/pathogens15101021 (PMC13708061; doi:10.3390/pathogens15101021)
Supplement: Supplementary file 1 [file pathogens-15-01021-s001.zip › Supplementary Table S2.pdf]

Supplementary Table S2. Inflammatory and apoptotic biomarker profiles by combined HIV/HCV status.

Cytokine/chemokine Median (Q1-Q3)	HIV- & HCV- (n = 10)	HIV+ & HCV- (n = 39)	HIV- & HCV+ (n = 18)	HIV+ & HCV+ (n = 22)	Kruskal-Wallis p-value (FDR adj p)
IL-6 pg/mL	3.340 (0.936-4.395)	3.140 (1.760-8.840)	9.940 (3.460-26.525)	3.923 (0.851-6.780)	0.035* (0.046)
IL-8 pg/mL	3.095 (2.295-5.280)	6.688 (3.910-15.855)	11.073 (6.130-32.925)	8.060 (3.545-17.510)	0.005* (0.033)
SDF1 alpha	675.840 (585.590-701.730)	777.053 (684.500-952.185)	775.478 (635.505-929.630)	826.170 (621.205-976.845)	0.087 (0.100)
Mip-1 alpha	5.170 (3.653-7.250)	7.470 (4.002-14.490)	13.035 (3.653-22.395)	7.758 (4.002-12.410)	0.231 (0.231)
Mip-1beta	15.760 (12.260-17.555)	19.440 (15.060-28.685)	29.688 (25.625-33.945)	20.958 (15.660-30.340)	0.011* (0.033)
RANTES	258.710 (174.890-361.895)	383.933 (171.220-563.255)	111.830 (58.415-349.065)	264.880 (151.470-681.215)	0.029* (0.046)
TNFalpha	8.380 (6.385-10.560)	11.680 (8.900-23.335)	16.763 (11.560-41.830)	17.290 (9.640-22.430)	0.016* (0.033)
M30 U/L	97.510 (73.783-118.935)	89.383 (69.532-108.080)	115.312 (95.007-274.712)	90.197 (73.884-181.733)	0.013* (0.033)

Q1-Q3, first to third quartile (interquartile range); FDR, false discovery rate. p-values are from the four-group Kruskal-Wallis test; FDR-adjusted p-values were computed by the Benjamini-Hochberg procedure across the 8 analytes. * $p < 0.05$.
